# A case report of isolated distal upper extremity weakness due to cerebral metastasis involving the hand knob area

**DOI:** 10.1186/s12885-018-4857-9

**Published:** 2018-10-03

**Authors:** András Folyovich, Viktória Varga, György Várallyay, Lajos Kozák, Mária Bakos, Erika Scheidl, Katalin Anna Béres-Molnár, Zita Kajdácsi, Dániel Bereczki

**Affiliations:** 1 Department of Neurology and Stroke Center, Szent János Hospital, Budapest, Hungary; 2 Department of Neurology, Uzsoki Hospital, Budapest, Hungary; 3 MR Research Centre, Semmelweis University, Budapest, Hungary; 4 Department of Diagnostic Radiology, Szent János Hospital, Budapest, Hungary; 5 Korányi National Institute for Tuberculosis and Pulmonology, Budapest, Hungary; 6 Department of Neurology, Semmelweis University, Balassa u. 6., H- 1083, Budapest, Hungary

**Keywords:** Isolated distal upper extremity weakness, Hand knob, Brain metastasis

## Abstract

**Background:**

Unilateral weakness of an upper extremity is most frequently caused by traumatic nerve injury or compression neuropathy. In rare cases, lesion of the central nervous system may result in syndromes suggesting peripheral nerve damage by the initial examination. Pseudoperipheral hand palsy is the best known of these, most frequently caused by a small lesion in the contralateral motor cortex of the brain. The ‘hand knob’ area refers to a circumscribed region in the precentral gyrus of the posterior frontal lobe, the lesion of which leads to isolated weakness of the upper extremity mimicking peripheral nerve damage. The etiology of this rare syndrome is almost exclusively related to an embolic infarction.

**Case presentation:**

We present the case of a 70-year-old male patient with isolated left sided upper extremity weakness and clumsiness without sensory disturbance suggesting a lesion of the radial nerve. Nerve conduction studies had normal results excluding peripheral nerve damage. Neuroimaging (cranial CT and MRI) detected 3 space occupying lesions, one of them in the right precentral gyrus. An irregularly shaped tumor was found by CT in the left lung with multiple associated lymph node conglomerates. The metastasis from this mucinous tubular adenocarcinoma with solid anaplastic parts to the ‘hand knob’ area was responsible for the first clinical sign related to the pulmonary malignancy.

**Conclusions:**

Pseudoperipheral palsy of the upper extremity is not necessarily the consequence of an embolic stroke. If nerve conduction studies have normal results, neuroimaging – preferably MRI – should be performed, as lesion in the hand-knob area of the precentral gyrus can also be caused by a malignancy.

## Background

Isolated upper extremity weakness is predominantly attributed to an injury of the peripheral nervous system. Weakness resulting from damage of the radial, ulnar, or median nerve are most commonly due to traumatic injury or compression neuropathy; however, it may develop as a symptom in association with other conditions including amyotrophic lateral sclerosis, cervical radiculopathy, or thoracic outlet syndrome. Isolated weakness of the upper extremity is rare in central nervous system damage. In such cases lesions of the motor cortex or of the downstream corticospinal tract are responsible for the clinical signs. The ‘hand knob’ area is a circumscribed region of the motor cortex in the precentral gyrus of the posterior frontal lobe [1], the lesion of which leads to isolated weakness of the contralateral upper extremity mimicking peripheral nerve damage. This rare symptom is almost exclusively related to an underlying cortical infarction. The lesion presents in a misleading symptomatology, raising the suspicion of peripheral neuropathy, which may pose a challenge in the differential diagnosis.

Pseudoperipheral paresis of the upper extremity is known as a rare stroke syndrome. Cortical hand knob strokes represent less than 1% of all hospitalized ischemic strokes in single center surveys. The reported rate was 8 out of 815 (0.98%) in a Turkish study over 1,5 years [2], whereas a 5-year survey in Germany [3] found that 29 out of 3499 patients (0.8%) had a stroke of the cortical hand knob area. In a 5-year study in Norway, out of 866 patients with acute ischemic stroke 6 (0.7%) had a single lesion in the hand knob region [4]. In our study of 25 cases over 10 years we estimated the rate of hand knob strokes to be 0.35% [5]. Although the hand knob area certainly may be affected by intracerebral tumors like glioblastoma (see Fig. 1 of Krieg et al. [6]), the incidence of malignant tumors in the background of isolated hand knob lesions have not been established.

**Fig. 1:**
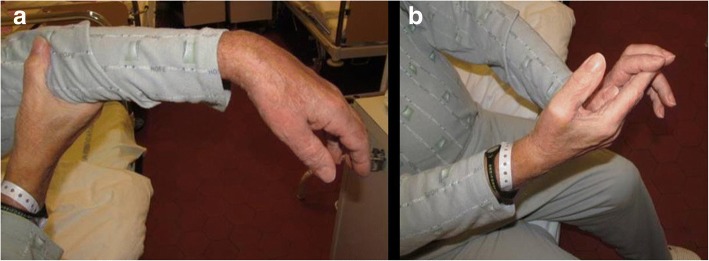
Isolated central weakness of the left upper extremity. Wrist drop (**a**) and decreased handgrip strength (**b**)

## Case report

**Fig. 2:**
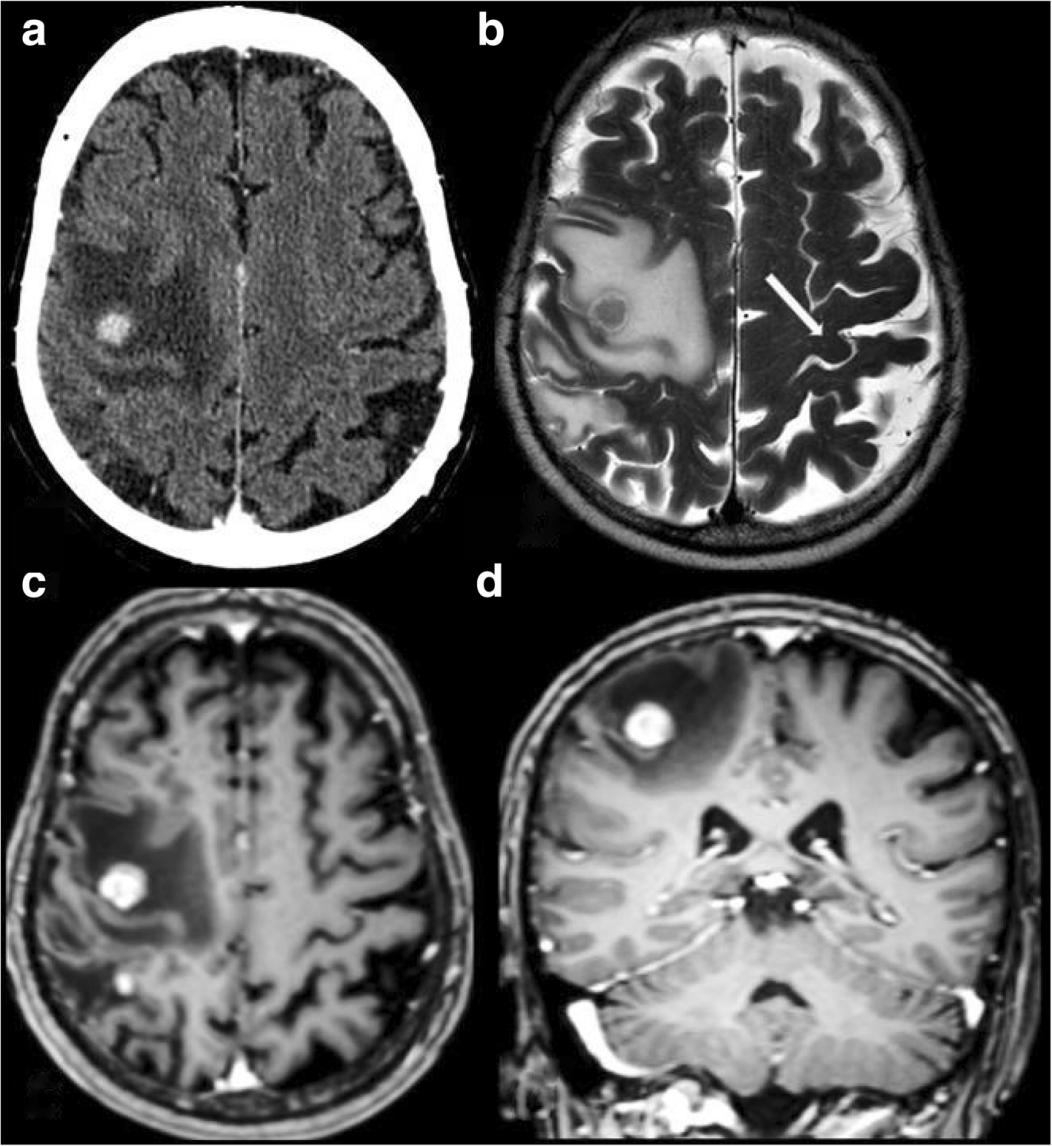
Contrast-enhanced axial CT (**a**), T2W axial MR (**b**), contrast-enhanced T1W axial (**c**) and coronal (**d**) MR. Intensive contrast enhancement (11 mm) in the right precentral gyrus, corresponding to the ‘hand knob’s area, with large perifocal edema. The left ‘hand knob’ is normal (arrow). Four other smaller enhancing metastases in the brain (not shown)

**Fig. 3:**
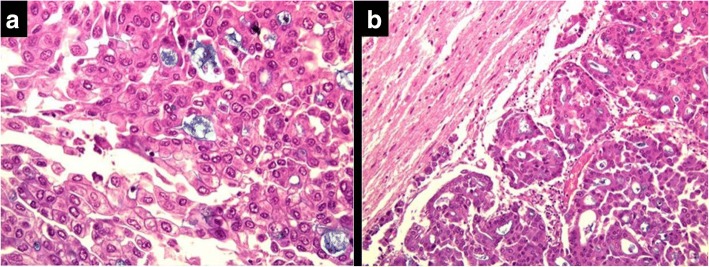
Pulmonary malignancy (mucinous tubular adenocarcinoma with anaplastic solid parts), 400× magnification, Hematoxylin & Eosin + alcian blue (**a**). Cerebral metastasis (mucinous tubular adenocarcinoma with anaplastic solid parts), 200×, magnification, Hematoxylin & Eosin + alcian blue (**b**)

The 70-year-old male patient was admitted to the Department of Neurology with isolated left upper extremity weakness and clumsiness. He had wrist drop and was unable to grip with the fingers. He complained of no sensory loss or numbness. His past medical history included long-term oral anticoagulation due to recurrent lower extremity deep vein thrombosis, glaucoma, and a non-significant aortic valve stenosis, with vascular risk factors including a 5-year history of treated hypertension, hypercholesterolemia, and a history of non-significant stenosis of the left anterior descendent coronary artery. On admission, no abnormality could be detected by physical examination, apart from the neurological signs, which included a wrist drop on the left side with decreased grip strength (Fig. 1). Pronation and wrist dorsiflection were lost, as well as the ability to form a ring with the thumb and the index. The reflexes of the left upper extremity were slightly brisk, with not pathological reflexes present and no sensory deficits. Laboratory parameters were without abnormal findings except for a slightly elevated fasting blood glucose level. Cranial CT revealed contrast-enhancing, irregularly shaped lesions with diameters of 7, 10, and 9 mm (in the temporal, parietal, and frontal lobes, respectively), surrounded by perifocal edema. Carotid duplex ultrasonography did not demonstrate signs of focal atherosclerotic plaques, circulatory disturbance or stenosis on either side. In accordance with the findings of the CT scan, the cranial MRI confirmed the tumor in the right precentral gyrus, corresponding to the ‘hand knob’, accompanied by further similar lesions posteriorly in the parietal and the temporal lobes, and in the left frontal lobe (Fig. 2). Electromyography and nerve conduction studies did not support a peripheral origin underlying the paresis. The neuropsychological examination revealed a deficit in the Luria three-step test as a single alteration, which performance could, however, be remarkably improved by verbal clues. Chest X-ray performed as part of the search for primary tumor revealed no abnormality. Non-contrast and contrast-enhanced chest CT detected an irregularly shaped mass in segment 10 of the left lung, with inhomogeneous enhancement of the contrast agent and multiple associated lymph node conglomerates, suggesting lung cancer as the primary tumor. Abdominal ultrasonography did not detect malignancy. The patient received palliative steroid therapy, with no improvement in the paresis during the observation period. Based on the recommendation of the tumor board, the patient was transferred to the Department of Pulmonology for bronchoscopic tissue sampling. The verification of the diagnosis by this means, however, was unsuccessful. The diagnostic process had to be suspended due to an acute bleeding duodenal ulcer, and the patient received blood transfusion and local hemostatic treatment in the Department of Surgery. In association with the antibiotic treatment, the patient developed pseudomembranous colitis caused by Clostridium difficile. In addition to the persisting colitis, fever occurred accompanied by hemoptysis and coughing. The subsequent chest X-ray did not confirm pneumonia. Despite the applied antibiotic and oxygen therapy, respiratory failure developed, and the patient passed away 2 months after the onset of the neurological symptom. The autopsy and the histopathological analysis identified an adenocarcinoma (mucinous tubular adenocarcinoma with solid anaplastic parts) both in the brain and the lung; (Fig. 3).

## Discussion and conclusions

Isolated upper extremity weakness is rarely caused by a lesion localized to the central nervous system. As the clinical signs suggest peripheral neuronal disease, the usual workup of such patients starts with nerve conduction studies. With normal results of the electrophysiological tests, another etiology should be searched. An injury to a circumscribed territory of the precentral gyrus (primary motor cortex) referred to as ‘hand knob’ results in clinical signs mimicking peripheral nerve lesion that is called “pseudoperipheral” palsy. According to the Penfield-Rasmussen map, primary motor neurons innervating the upper extremity are localized in the lower third of the dorsolateral surface of the precentral gyrus [1, 7]. This region of the precentral gyrus is a knob-like structure, reminiscent of an epsilon or an omega in the axial functional MRI scans, whereas resembling a hook in the sagittal scans [8]. The etiology behind this rare symptom is almost exclusively reported to be a cortical infarction [3, 9–14], with hardly any reference to alternative causes [15]. Intraoperative cortical stimulation, functional MRI and navigated transcranial magnetic stimulation studies clearly identified the region in the precentral gyrus of the posterior frontal lobe which is associated with hand movements, therefore should be saved during neurosurgical interventions, like resections of primary and metastatic brain tumors in the perirolandic area [6, 16, 17].

Our case confirms that circumscribed damage to the hand knob resulting in isolated distal upper extremity weakness may be caused not only by cerebral ischemia but also by a brain metastasis. The isolated weakness of the upper extremity was the first and only symptom that initiated the diagnostic process, eventually leading to the diagnosis of a primary pulmonary malignancy. The clinical diagnosis was confirmed by autopsy and histopathological examination.

Although it has been suggested that patients with isolated hand palsy without alternative explanations for peripheral damage should be aggressively treated for acute ischemic stroke [18], pseudoperipheral palsy of the upper extremity is not necessarily the consequence of an ischemic cerebrovascular lesion. If nerve conduction studies have normal results, neuroimaging should be performed, as a lesion in the hand-knob area of the precentral gyrus can also be caused by a malignancy affecting this region. As CT misses the hand knob lesion in a considerable rate, MRI should be the preferred neuroimaging method.
